# Comment on: Cannula complications using elastomeric infusers in Hospital in the Home

**DOI:** 10.1093/jacamr/dlaa088

**Published:** 2020-10-22

**Authors:** Hugh G Dickson, Evan Alexandrou, Jayanthi Ramanathan, Dana West

**Affiliations:** 1 Ambulatory Care, Liverpool Hospital, South Western Sydney Local Health District, Liverpool, Australia; 2 The University of New South Wales, Kensington, NSW, Australia; 3 Western Sydney University, Sydney, NSW, Australia

Sir,

Ryan *et al*.^1^ describe a randomized controlled trial examining the rate of short peripheral cannula (SPC) complications between 44 patients receiving bolus doses of IV cefazolin 2 g twice daily and 60 patients receiving cefazolin 6 g daily via elastomeric pump.^1^ There are a number of deficiencies in the paper, most of which stem from a lack of adherence to the CONSORT guidelines, which are clearly referred to in the journal’s instructions for authors. For example, the trial is open label but does not explicitly state this or discuss bias from the lack of blinding. There is no statement about whether the trial was analysed on an ITT basis or on a PP basis, which makes the rigour of the trial impossible to assess. In addition, no information is provided about the number of patients who were screened, considered eligible and not randomized, and the number of patients randomized and then not included in the final analysis, again a major requirement for assurance that the trial was conducted with rigour. Significantly, no CONSORT flow chart has been provided. 

As regards random allocations, these were not computer generated but rather by toss of a coin, which can pose issues with concealment prior to randomization and selection bias. We could not find any details on sample size calculation. Numbers in each arm were chosen so as to be consistent with ‘similar research’. This does not substitute for a formal power calculation. A difference of 13% between the groups was found for the need for additional cannulation, with 25% of the bolus group needing one additional cannula and 38% of the infuser group requiring at least one additional cannula. The difference between the groups regarding additional cannulation was non-significant. However, the approximate sample size required via nomogram to detect this 13% difference with 90% power using a cut-off for statistical significance of 0.05 is 250 in each arm.^2^ The trial therefore is grossly underpowered.

The methodology around cannula placement is not uniform, which allows another source of bias. Some patients had their initial cannulation by emergency department staff and the rest by the nursing staff in the Hospital in the Home programme. The exact breakdown should have been provided, as there is a higher risk of phlebitis in cannulas inserted in emergency departments.^3^

Problems with methodology extend to the reporting of complications. Given that the complication rate is associated with catheter size,^4^ the exact breakdown of all catheter-associated complications between groups should have been provided. The nurses assessing the patient for signs of phlebitis were provided with a visual phlebitis scale so as to standardize reporting. No information is given about the results from use of this system. We do not know, for example, whether the numbers of patients with more severe signs of phlebitis differed between groups. We do not know if the site of cellulitis differed between groups, and we are left to guess at whether the outcome of treatment was the same between the two groups.

The method by which patency of the cannula was determined is not described. We do not know how the blockage in patients receiving infuser therapy was assessed. ‘No-flow’ events through an elastomeric infuser can be associated with infuser valve failure or, more commonly in our experience, failure to fully engage a needleless connector. The cannula can be patent, but the infuser will not flow in these situations.

In order to properly compare the two groups for differences between SPC complication rates, the duration of therapy and thus dwell times should have been standardized so that dwell times were identical between the groups. However, there is a difference between the two groups, with the infuser group averaging 5.75 days of treatment and the SPC group averaging 4.75 days. The SD or range of SPC dwell times of SPC is not reported. There is no information about the standard policy in the service for dwell time of SPC before replacement and no information about the speed and method of administration of the bolus doses of cefazolin, all factors that might influence the need to replace a cannula.^5^ Although gauge size of SPC is described, no information is provided on the length of SPC or whether extension sets were used or the type of dressing and securement technique—all of which can influence SPC survival rates.^4^^,^^6^ Table 4, which reports the complication results, has incorrect data entries, with the numbers in the first section not consistent with the next two sections.

Given that the antimicrobial action of cefazolin depends on the fraction of the time during the dosing interval in *fT*_>MIC_ for the pathogenic bacteria,^7^ and a steady-state concentration of cefazolin with infusion takes 6–10 h to achieve, we would have expected a loading dose to be administered prior to the connection of the infusion. An IV bolus dose of 2 g was required at some time prior to enrolment in the study, but the time between this dose and the subsequent commencement of the infusion is not given. As the pharmacokinetics of cefazolin is dependent on the renal function of the patient, we would have expected the renal function to have been examined to ensure equivalence between the groups.

Finally, there is no statement in the paper that the trial was registered in any database of clinical trials despite this being a requirement of the journal for authors of clinical trials. Overall, we would not be prepared on the basis of the study to recommend a change to the conventional practice of use of a midline or central venous catheter for delivery of antimicrobial therapy via an elastomeric infuser.

## Funding

This study was carried out as part of our routine work.

## Transparency declarations

None to declare.

## References

[1] Ryan D Miller J Campbell J. Cannula complications using elastomeric infusers in Hospital in the Home. JAC-Antimicrob Resist 2020; 2: dlaa033. 10.1093/jacamr/dlaa033 PMC8210130 34223000

[2] Whitley E Ball J. Statistics review 4: sample size calculations. Crit Care 2002; 6: 335–41. 12225610 10.1186/cc1521 PMC137461

[3] Guembe M Perez-Granda MJ Capdevila JA et al Nationwide study on peripheral-venous-catheter-associated-bloodstream infections in internal medicine departments. J Hosp Infect 2017; 97: 260–6. 28716670 10.1016/j.jhin.2017.07.008

[4] Alexandrou E Ray-Barruel G Carr PJ et al Use of short peripheral intravenous catheters: characteristics, management, and outcomes worldwide. J Hosp Med 2018; 13: doi:10.12788/jhm.3039. 10.12788/jhm.3039 29813140

[5] Keogh S Mathew S. Peripheral Intravenous Catheters: A Review of Guidelines and Research. ACSQHC, 2019.

[6] Corley A Ullman AJ Mihala G et al Peripheral intravenous catheter dressing and securement practice is associated with site complications and suboptimal dressing integrity: a secondary analysis of 40,637 catheters. Int J Nurs Stud 2019; 100: 103409. 31629208 10.1016/j.ijnurstu.2019.103409

[7] Bellouard R Deschanvres C Deslandes G et al Population pharmacokinetic study of cefazolin dosage adaptation in bacteremia and infective endocarditis based on a nomogram. Antimicrob Agents Chemother 2019; 63: e00806-19. 31307987 10.1128/AAC.00806-19 PMC6761522

